# Procedural risk factors for deep and organ/space surgical site infection post-coronary artery bypass graft surgery

**DOI:** 10.1017/ash.2024.374

**Published:** 2024-09-09

**Authors:** Abarna Pearl, Patrick S. Gordon, Baevin S. Feeser, Dana E. Pepe, Preeti Mehrotra, Sharon B. Wright

**Affiliations:** 1 Division of Infectious Diseases, Beth Israel Deaconess Medical Center, Boston, MA, USA; 2 Division of Infection Control/Hospital Epidemiology, Beth Israel Deaconess Medical Center, Boston, MA, USA

## Abstract

In this study, we examined the impact of the number and type of arterial grafts, and surgical dressing type, on deep and organ/space surgical site infection following coronary artery bypass graft procedures. Bilateral internal mammary artery grafts and negative pressure wound therapy were associated with higher odds of infection.

## Introduction

Advances in coronary artery bypass graft (CABG) techniques have improved graft patency and survival, however, little is known about the impact of the number and type of arterial grafts used on post-CABG surgical site infections (SSIs).^1^ Literature suggests that even for the same number of arterial grafts used, specific artery types may impact SSI incidence. Surgical dressing method, ie surgical adhesive versus negative pressure wound therapy (NPWT), may also impact SSI risk. Previously, we found no difference in SSI incidence related to harvest technique (skeletonized versus pedicled) or use of single versus bilateral internal mammary (BIMA) graft.^2^ Here we explore modifiable procedural SSI risk factors, including artery type and surgical dressing method.

## Methods

We conducted a retrospective cohort study of patients over 18 years old who underwent CABG at an academic tertiary care center from 1/2019 through 12/2022. Variables abstracted from hospital data marts and the Division of Cardiac Surgery database included demographics, comorbidities, surgical technique, surgical dressing, and surgeon. Post-CABG deep and organ/space SSIs occurring within 90 days were identified by infection preventionists during routine surveillance using National Healthcare Safety Network definitions.^3^

Surgical dressing was recorded as “NPWT,” “surgical adhesive,” “missing” or “both.” Data were validated by review of provider notes of a random sample of 10% of the entire cohort. Sensitivity analyses regarding the effect of NPWT on the odds of SSI were performed, classifying patients with “missing” or “both” dressings into other categories or in separate categories.

Univariate associations between exposures and SSI were analyzed using Wilcoxon’s rank sum for continuous variables and Fisher’s exact or Chi-square tests for categorical variables. A sub-analysis evaluated the risk of SSI in patients with two arterial grafts by type of artery used, specifically BIMA versus single internal mammary artery plus radial (SIMA-Radial) graft. Exploratory univariate analyses were conducted to identify potential confounding variables between SSI and arterial graft type and between SSI and surgical dressing method.

Propensity score logistic regression was used to find the adjusted effect of NPWT on the odds of SSI, with diabetes mellitus (DM), smoking status, age > 75, body mass index (BMI) > = 30, and sex as input variables for the propensity score.

SAS 9.4 was used for all statistical analyses. Extreme values of BMI were excluded from all analyses (e.g., BMI > 70 (5 patients) or BMI < 13 (11 patients)).

## Results

Of the 2050 included patients, 23 developed an SSI (12 deep and 11 organ/space). Characteristics of patients with and without SSI are shown in Table 1. In univariate analyses, DM (*P* < .001), NPWT (*P* < .001), and longer case duration (*P* = .04) were associated with SSI. There was no significant association between SSI and the total number of arterial grafts, combination of grafts used, harvest technique, surgeon, or surgery year.

**Table 1.** Select characteristics of coronary artery bypass graft (CABG) patients with and without surgical site infection (SSI)

Variable	Deep or organ/space SSI^a^, n = 23 n (%)^b^	No deep or organ/space SSI^a^, n = 2027 n (%)^b^	*P* value^c^
**Male sex**	16 (69.6)	1646 (81.2)	0.18
**Age (years)** ^d^	62 (55–73)	68 (61–74)	0.1
**Diabetes mellitus**	19 (82.6)	933 (46.0)	**0.0005**
**Smoking**	14 (60.8)	1111 (55.0)	0.68
**BMI** ^e^			0.66
BMI<30	17 (73.9)	1371(68.2)	
BMI≥30	6 (26.1)	640(31.8)	
**Procedure type**			0.32
CABG**^f^**	16 (69.6)	1584 (78.2)	
CABG with other cardiac procedure	7 (30.4)	443 (21.9)	
**Harvest technique**			1
Pedicle	14 (1.2)	1151 (98.8)	
Skeletonized	9 (1.1)	806 (98.9)	
**Arterial grafts used** ^g^			0.14
Veins only	0	59 (100)	
Radial artery only	0	6 (100)	
SIMA only	15 (1.2)	1253 (98.8)	
SIMA + Radial only	0	281 (100)	
BIMA only	4 (2.6)	150 (97.4)	
BIMA + Radial only	4 (1.4)	278 (98.6)	
**Surgical dressing**			**<0.0001**
NPWT**^h^**	18 (78.2)	558 (27.5)	
Surgical adhesive	5 (21.7)	1469 (72.5)	
**Case duration (mins)** ^d^	249 (212–291)	223 (188–270)	**0.04**

a Surgical site infection.

b Column percentages are shown.

c Fisher’s exact or Chi-square for categorical variables; Wilcoxon’s rank sum for continuous variables.

d Variable expressed as median and interquartile range.

e Body mass index.

f Coronary artery bypass graft.

g With or without venous grafts.

h Negative pressure wound therapy.

Among patients with two arterial grafts, 281 patients underwent SIMA-Radial and 154 underwent BIMA. None of the SIMA-Radial versus four of the BIMA, developed SSI (*P* = .02). In the BIMA group, there was a greater proportion of skeletonized (versus pedicled) arterial harvest (OR 2.19, *P* = .016). These patients also had younger ages (63 vs 68 years, *P* < .001), lower BMI (26.6 vs 27.8, *P* = .02), and longer case duration (238 vs 217 minutes, *P* = .003).

On validation using provider notes, 90% of the patients with “both” dressings were found to have NPWT, therefore these patients were included in the “NPWT” category. For patients with “surgical adhesive” recorded, there was no mention of the dressing type in 63% of patients. Similarly, on review of patients with “missing” dressing, 42% had neither dressing type recorded and 30% had surgical adhesive listed. Surgical adhesive likely represents the default and therefore may not be specifically recorded. Thus, patients found to have “missing” dressing were categorized as having “surgical adhesive” in the analysis.

In total, 576 and 1474 patients of the total were categorized as “NPWT” (376 “NPWT”; 200 “both”) versus “surgical adhesive” (1231 “surgical adhesive”; 243 “missing”) respectively. The odds ratio (OR) of SSI in patients with NPWT versus surgical adhesive was 9.48 (3.50–25.65). The OR of SSI remained significantly higher in patients with NPWT compared to those with surgical adhesive in all sensitivity analyses. In exploratory analyses of characteristics in patients with NPWT versus surgical adhesive, patients with female sex, DM, and BMI ≥ 30 were more likely to receive NPWT dressings. Patients with NPWT also had a longer median case duration. There was no significant association between dressing type and either smoking status, number of arteries used, combination of arterial grafts used, or surgical year. Table 2 shows select patient characteristics by surgical dressing type.

**Table 2.** Select characteristics of coronary artery bypass graft (CABG) patients, according to surgical dressing type

Variable	NPWT^a^ n = 576 (n,%)^b^	Surgical adhesive dressing n = 1474 (n,%)^b^	*P* value^c^
**Sex**			**<0.0001**
Male	366 (22.0)	1296(78.0)	
Female	210 (54.1)	178 (45.9)	
**Age (years)**			0.17
≤50	42 (35.3)	77 (64.7)	
50–75	408 (27.3)	1085 (72.7)	
>75	126 (28.8)	312 (71.2)	
**Diabetes mellitus**			**<0.0001**
Yes	363 (38.1)	589 (61.9)	
No	213 (19.4)	885 (80.6)	
**BMI** ^d^			**<0.0001**
<30	265(19.1)	1123 (80.9)	
≥30	306 (47.4)	340 (52.6)	
**Case duration (mins)** ^e^	233 (195–282)	221 (186–265)	**<0.0001**

a Negative pressure wound therapy.

b Row percentages are shown.

c Fisher’s exact or Chi-square for categorical variables; Wilcoxon’s rank sum for continuous variables.

d Body mass index.

e Variable expressed as median and interquartile range.

In the propensity score regression, the adjusted OR of SSI in those who had NPWT compared to surgical adhesive was 10.45 (3.62–30.16).

## Discussion

The post-CABG SSI rate was 1.1%, which is within the expected range.^4^ Our study found no significant association between post-CABG SSI and the number of arterial grafts or harvest technique. Amongst a subset of CABG patients who had two arterial grafts, those with BIMA were more likely to have SSI than those with SIMA-Radial. Additionally, NPWT was associated with a higher odds of SSI than surgical adhesive.

BIMA graft has been associated with a higher risk of SSI than SIMA in randomized controlled trials.^5^ This could be related to artery location and increased de-vascularization of the sternum when both internal mammary arteries are used.

It is unclear why NPWT was associated with a higher odds of SSI than surgical adhesive. NPWT has historically been associated both with reduction in SSIs when used prophylactically on surgical wounds and with improved outcomes after post-sternotomy mediastinitis, compared to standard surgical dressing and closure methods.^6–9^ At our institution NPWT is typically applied after closure of the sternotomy wound in patients deemed to be at high SSI risk using a literature-based scoring system including age, sex, extreme BMI, DM, smoking, and other comorbidities.^4^ Thus, our results could be due to confounding by indication, whereby those who are selected by surgeons to have NPWT are inherently at higher risk for SSI. However, further investigation via propensity score regression to account for potential confounding did not support this hypothesis. This may be explained by additional factors considered by surgeons when deciding to employ NPWT (eg, structural factors observed during procedure like sternal thickness), not captured in our study.^10^ Variability in the application of NPWT, including establishment of a secure seal, may also affect its effectiveness in SSI prevention.

Limitations of our study include the inability to perform multivariable regression to simultaneously adjust for multiple confounding variables, due to the low number of deep and organ/space SSIs. For surgical dressing type, confounding was partly overcome using propensity score regression, which adjusts for known factors for choosing NPWT in a single, composite covariate. Additionally, the surgical dressing data may have been subject to misclassification, though a sample was validated and sensitivity analyses were performed. In these analyses, incorporation of “missing” data in the “NPWT” category only strengthened our findings.

Overall, potentially modifiable procedural risk factors, including use of BIMA and surgical dressing type, could affect the odds of SSI post-CABG surgery. An unexpected finding was the higher SSI odds with use of NPWT in our population. Additional research across multiple centers, including prospective collection of reasons for surgeon dressing selection, would be important to further understand our results.
